# Low-Intensity Pulsed Ultrasound Alleviates Hypoxia-Induced Chondrocyte Damage in Temporomandibular Disorders by Modulating the Hypoxia-Inducible Factor Pathway

**DOI:** 10.3389/fphar.2020.00689

**Published:** 2020-05-14

**Authors:** Tao Yang, Chao Liang, Lei Chen, Jun Li, Wei Geng

**Affiliations:** ^1^Department of Dental Implant Center, Beijing Stomatological Hospital, School of Stomatology, Capital Medical University, Beijing, China; ^2^Department of Orthodontics, School of Stomatology, Shandong University, Jinan, China; ^3^Shandong Provincial Key Laboratory of Oral Tissue Regeneration, School of Stomatology, Shandong University, Jinan, China; ^4^Beijing Key Laboratory of Tooth Regeneration and Function Reconstruction, School of Stomatology, Capital Medical University, Beijing, China

**Keywords:** chondrocyte, hypoxia, low-intensity pulsed ultrasound (LIPUS), hypoxia-inducible factors (HIFs), temporomandibular joint disorder (TMD)

## Abstract

Temporomandibular disorders are a common cause of chronic pain in the orofacial region and have a complex and multi-factorial pathophysiology. Mechanical loading or inflammatory conditions have been shown to decrease oxygen tension within the joint cartilage and activate the hypoxia-inducible factor (HIF) pathway, which in turn aggravates the pathological processes underlying temporomandibular joint (TMJ) disorders. We previously showed that low-intensity pulsed ultrasound (LIPUS) treatment effectively repairs TMJ injury induced by chronic sleep deprivation (CSD). Here, we explored the effects of LIPUS treatment on hypoxia-induced chondrocyte injury. We found that it effectively restored the proliferation capacity of mandibular chondrocytes under hypoxic conditions and lowered their rate of apoptosis. Chondrogenic capacity, as assessed by type II collagen levels, and mucin-positive areas were also significantly increased after LIPUS treatment. Levels of matrix metalloprotein-3 and interleukin-6 decreased in mandibular chondrocytes following this treatment, whereas the expression of tissue inhibitor of metalloproteinase-1 increased. We also found that HIF-1α expression was upregulated in mandibular chondrocytes under hypoxic conditions and was further enhanced by LIPUS treatment. Similarly, HIF-2α levels increased in mandibular chondrocytes under hypoxic conditions but decreased following LIPUS treatment. Subsequently, we established a CSD-induced TMJ injury model and found that LIPUS increased mucin-positive areas as well as HIF-1α expression and decreased HIF-2 level in the chondrocyte layer. Together, our results indicate that the protective effect of LIPUS on chondrocyte is partly associated with the HIF pathway.

## Introduction

Temporomandibular disorders (TMDs) affect approximately 6–12% of the population (Lipton et al., 1993; Brett et al., 2018) and manifest as pain in the mastication muscles and temporomandibular joint (TMJ), with associated joint noise when opening or closing the mouth (Scrivani et al., 2008; Wadhwa and Kapila, 2008). These disorders can occur due to muscle hyperfunction or parafunction, trauma, or hormonal and/or articular changes (Scrivani et al., 2008; Liu and Steinkeler, 2013). Approximately 80% of patients with TMD suffer from clinical conditions such as disc displacement, arthralgia, osteoarthrosis, and osteoarthritis (Warren and Fried, 2001; Wadhwa and Kapila, 2008). TMDs have a complex and multifactorial etiology (Florjanski et al., 2019). Mechanical loading has been shown to contribute to their onset and progression (Holyoak et al., 2019), and metabolism-related dysbiosis and the accumulation of inflammatory cytokines might also be involved in disease pathogenesis (Wadhwa and Kapila, 2008; Park and Chung, 2016). However, the precise mechanisms underlying TMDs remain unclear.

Hypoxia is a common pathophysiological condition associated with TMDs. Although articular cartilage exists at low oxygen tension (~5%) in its normal physiological state (Zhou et al., 2004), the oxygen tension of mandibular condylar cartilage can decrease when mechanical loading exceeds the adaptive capacity of the joint or when it is exposed to inflammatory conditions (Huang et al., 2017). Multiple signaling pathways are activated in response to hypoxia, including the hypoxia-inducible factor (HIF) pathway, which regulates metabolism, cell death, and survival (Semenza, 2012; Zhang et al., 2017). Hypoxia has also been shown to decrease type II collagen levels and reduce synthesis of extracellular matrix in porcine articular cartilage (Cisewski et al., 2015). In addition, hypoxia directly regulates the secretion of vascular endothelial growth factor (VEGF) and inflammatory cytokines such as interleukin (IL)-6 (Arjamaa et al., 2017). These inﬂammatory mediators can upregulate matrix metalloproteinase (MMP) expression and decrease tissue inhibitor of metalloproteinase (TIMP) production, resulting in degradation of the extracellular matrix and chondrocyte apoptosis (Ying et al., 2017; Sun et al., 2019).

Currently, TMD therapy involves jaw exercise, ultrasound, and acupuncture (Armijo-Olivo et al., 2016). Treatment mainly focuses on relieving pain and improving TMJ biofunctions; however, these effects tend to be reversible and conservative (Mitsui et al., 2016). Low-intensity pulsed ultrasound (LIPUS), which uses a low frequency (1–3 MHz) and an intensity less than 100 mW/cm^2^ (Nagao et al., 2017), exerts pleiotropic biological effects *via* its mechanical actions and weak thermal effects (Vaughan et al., 2010). LIPUS has been used as a safe and effective non-invasive therapy to promote fracture healing, resulting in bone regeneration, injured tissue repair, and decreased inflammation (Xin et al., 2016; Lou et al., 2017). In addition, it has a positive effect on chondrocyte restoration in cartilage explants and osteoarthritis (Uddin et al., 2016; Rothenberg et al., 2017). Thus, LIPUS might represent an effective treatment for hypoxia-induced chondrocyte injury.

We previously showed that LIPUS can inhibit chronic sleep deprivation (CSD)-induced condylar cartilage injury in a rat model by decreasing MMP-3/TIMP-1 and RANKL/OPG ratios (Liang et al., 2019). However, its biological effects on chondrocytes under hypoxic conditions and its impact on the HIF pathway remain unclear. In this study, we aimed to determine whether LIPUS can ameliorate hypoxia-induced chondrocyte injury by regulating the HIF pathway *in vitro*, in addition to confirming its beneficial effect using a rat model of CSD.

## Materials and Methods

### Rat Mandibular Condylar Chondrocyte Cell Culture

Our experiments were following standard biosecurity and institutional safety procedures with the guidance of professional experimental technician working in Department of Research Laboratory, Beijing Stomatological Hospital, School of Stomatology, Capital Medical University, Beijing, China.

Articular cartilage is an avascular tissue with a low oxygen tension of ~5% under normal physiological conditions (Hong et al., 2013). As inflammatory conditions can cause a gradual decrease in oxygen tension (Lee et al., 2016), we used 1% oxygen tension to establish our *in vitro* hypoxia model.

Chondrocytes were isolated from the mandibular condylar of 3-week-old male Wistar rats (SPF Biotechnology, Beijing, China) and identified using Toluidine blue staining (**Supplementary Figure 1**). Samples were soaked in PBS (Invitrogen, Carlsbad, CA, USA) containing 100 mg/ml penicillin and 100 mg/ml streptomycin (Invitrogen) for 1 min, washed thrice in PBS, and then minced fully. Samples were digested with 0.25% trypsin for 8 min and then digested with 0.2% collagenase II (C6885, Sigma, Germany) for 2 h. Tissues were cultured in Dulbecco's modified Eagle's medium supplemented with 20% fetal bovine serum (Invitrogen), 100 mg/ml penicillin, and 100 mg/ml streptomycin. The culture medium was changed every 3 d. Third passage (P3) chondrocyte cells were used in subsequent experiments and divided into three groups as follows: N group (normal group, cultured with 5% oxygen tension), L group (low oxygen tension group, cultured with 1% oxygen tension), L + LIPUS group (LIPUS treatment group, cultured with 1% oxygen tension).

### Animal Experiments

Eighteen 8-week-old male Wistar rats (SPF Biotechnology, Beijing, China) were randomly divided into three groups as follows: blank control group (without CSD treatment), CSD group, and CSD + LIPUS treatment group (LIPUS) (six animals per group). The CSD model was established as reported previously (Liang et al., 2019). All animals were maintained and fed under uniform conditions. Experiments were approved by the Ethics Committee of Beijing Stomatological Hospital (Approval code: KQYY-201610-001).

### LIPUS

An OSTEOTRON IV ultrasonic therapy device (ITO Ultrashort Wave Co. Ltd., Tokyo, Japan) was used for LIPUS treatment as follows: 45 mW/cm^2^ ultrasonic intensity, 1.0 MHz ultrasonic frequency, and 200 μs pulse width. A LIPUS probe, with a coupling gel at the bottom of the experimental plate, was employed for all *in vitro* experiments; chondrocytes were stimulated for 20 min per day. For *in vivo* studies, rats were treated with LIPUS (as described) for 4 weeks (20 min per day). The LIPUS probe was placed directly on the condyle skin. The diagram of the LIPUS equipment is shown in **Supplementary Figure 2**.

### Immunohistochemistry

After the animals were sacrificed, the TMJ was completely removed and fixed in 4% paraformaldehyde for 5 d, decalcified with 10% EDTA, and embedded in paraffin. Samples were sectioned into 5-μm slices, dehydrated with an alcohol gradient, and then incubated overnight at 4°C with the following primary antibodies: anti-HIF-1α (1:1,000, ab1, Abcam, Cambridge, England) and anti-HIF-2α (1:100, ab199, Abcam). The next day, the secondary antibody (1:200, ab97040, Abcam) was added and samples were incubated for 1 h at 25°C. Sections were observed using a microscope (OLYMPUS BX 61, Japan) and images were captured using a cellSens Standard. Six different sections were analyzed using Image-Pro Plus 6.0.

### Cell Viability and Apoptosis

For the cell viability assay, 5 × 10^3^ cells/well were plated in 96-well plates and cultured under different oxygen tensions. The L + LIPUS group was treated with LIPUS at a frequency of 20 min/d for 3 d. We used the standard CCK-8 assay (CK04, Dojindo, Japan) to determine cell proliferation at different time points. The CCK-8 reagent was added 12 h after LIPUS stimulation. The samples were incubated for 4 h before measuring the absorbance at 450 nm.

For the apoptosis assay, chondrocytes were seeded in six-well plates and cultured under different oxygen tensions. In the L + LIPUS group, stimulation lasted for 20 min per day. The ratio of apoptotic cells was detected 12 h after LIPUS stimulation. Apoptosis was detected using the Annexin V-FITC/PI Detection Kit (556547, BD Biosciences, USA), as per the manufacturer's instructions; Flow Jo 7.6 and GraphPad 6.0 were used to analyze the results.

### ELISA

Cellular supernatants were collected and centrifuged to remove particulate matter (300 g for 15 min at 4°C) before the IL-6 concentration was measured using the Rat IL-6 ELISA Kit (EK306/3-48, MultiSciences, Hangzhou, Beijing), as per the manufacturer's instructions.

### Alcian Blue Staining

For *in vitro* experiments, cells were seeded in six-well plates. Following treatment, cells were fixed with methanol, washed thrice with distilled water, and then stained with Alcian blue for 30 min. Images were observed using a fluorescent inverted microscope (IX71, OLYMPUS) and analyzed using Image-Pro Plus 6.0 (10 random fields per sample, in triplicate). For *in vivo* experiments, sections were dehydrated with an alcohol gradient, dyed with a 1% Alcian blue buffer (pH 2.5) for 10–15 min, and then observed using a microscope (OLYPUS BX 61, Japan). Images were captured using cellSens Standard and analyzed with Image-Pro Plus 6.0 (one random field per section, six different sections).

### Real-Time Quantitative Polymerase Chain Reaction (RT-qPCR)

Total RNA was obtained using TRIzol reagent (Invitrogen); then, 1 μg of total RNA was reverse transcribed with the PrimeScript RT Reagent Kit and gDNA Eraser (Takara, Shiga, Japan). RT-qPCR was carried out using the SYBR Premix Ex Taq II kit (Takara, Shiga, Japan) according to the manufacturer's protocol. The nucleotide sequences of the primers are shown in **Table 1** (Sangon Biotech, Shanghai, China). The relative expression of target genes was calculated by the ΔΔCt method.

**Table 1:** Primer sequences used for RT-qPCR.

Gene	Primer Sequence (5ʹ to 3ʹ)
*Col2*	Forward: AAGAGCAAGGAGAAGAAG
	Reverse: TTACAGTGGTAGGTGATG
*TIMP-1*	Forward: ACAGGTTTCCGGTTCGCCTAC
	Reverse: CTGCAGGCAGTGATGTGCAA
*MMP3*	Forward: TGGACCAGGGACCAATGGA
	Reverse: GGCCAAGTTCATGAGCAGCA
*Sox9*	Forward: GACGTGCAAGCTGGGAAAGT Reverse: CGGCAGGTATTGGTCAAACTC
*HIF-1α* *HIF-2α* *β-actin*	Forward: TCCAGTTACGTTCCTTTGATCAGT Reverse: TTCATCAGTGGTGGCAGTTG Forward: TCACTCATCCTTGCGACCAC Reverse: CAGGTGGCCGACTTAAGGTT Forward: ATGTGGATCAGCAAGCAGGA
	Reverse: GGTGTAAAACGCAGCTCAGTAA

### Western Blot Analysis

Total protein was harvested using RIPA buffer (R0278, Sigma) and the extracted protein concentration was determined *via* the Bradford method (Bio-Rad Laboratories, Hercules, CA, USA). The same amount of extracted protein was electrophoresed and separated on a 10% SDS polyacrylamide gel. Proteins were transferred to polyvinylidene fluoride membranes using a semi‐dry transfer system (Bio‐Rad), and then 5% skim milk was used to block non-specific binding for 2 h. The membranes were subsequently incubated overnight at 4°C with the following primary antibodies: rabbit polyclonal anti-Col 2 (1:3,000, ab34712, Abcam), rabbit polyclonal anti-MMP-3 (1:1,000, ab52915, Abcam), rabbit polyclonal anti-TIMP-1 (1:1,000; ab61224, Abcam), mouse monoclonal anti-HIF-1α (1:200; ab1, Abcam), rabbit polyclonal anti-HIF-2α (1:500, ab199, Abcam), mouse monoclonal anti-VEGF (1:200, ab1316, Abcam), β-actin antibody (1:100,000, AC026, ABclonal, Wuhan, China), and anti-HSP90 (1:1,000, ab13492, Abcam). Then, the membrane was incubated with the following horseradish peroxidase-conjugated secondary antibodies for 1 h at 25°C: goat anti-mouse secondary antibody (1:5,000, ab97040, Abcam) and goat anti-rabbit secondary antibody (1:2,000, ab97051, Abcam). Protein bands were visualized using Clarity Western ECL Substrate (170-5060, Bio‐Rad, USA) and analyzed using Image J.

### Immunofluorescence Staining

Cells were fixed in 4% paraformaldehyde for 20 min, permeabilized with 0.2% triton X-100 for 10 min, and then incubated with blocking buffer (ab126587, Abcam) for 1 h. Samples were incubated with primary antibodies (i.e. anti-Col 2; 1:200, ab34712, Abcam) overnight at 4°C and then with the secondary antibody (i.e. goat anti-rabbit IgG; 1:500, ab150080, Abcam) for 1 h at 25°C. DAPI was used as a nuclear counterstain. Samples were observed with a microscope (OLYMPUS, BX61) and analyzed with Image-Pro Plus 6.0 (three random fields per sample, in triplicate).

### Statistical Analysis

All data were analyzed using SPSS version 22.0 and presented as the mean ± standard deviation (SD), with a p-value < 0.05 considered significant (*p < 0.05, **p < 0.01, ***p < 0.001). All charts were made using GraphPad 6.0 and figures were generated using Photoshop CS 6.0. Data were analyzed for normality of distribution using the Shapiro-Wilk test and for homogeneity using the Bartlett test. One-way ANOVA was used to compare differences among three groups, and a *post-hoc* t-test was performed to analyze the differences between two groups. Each experiment was repeated at least three times.

## Results

### LIPUS Treatment Reduces Hypoxia-Induced Apoptosis in Mandibular Chondrocytes and Promotes Proliferation

**Figure 1:**
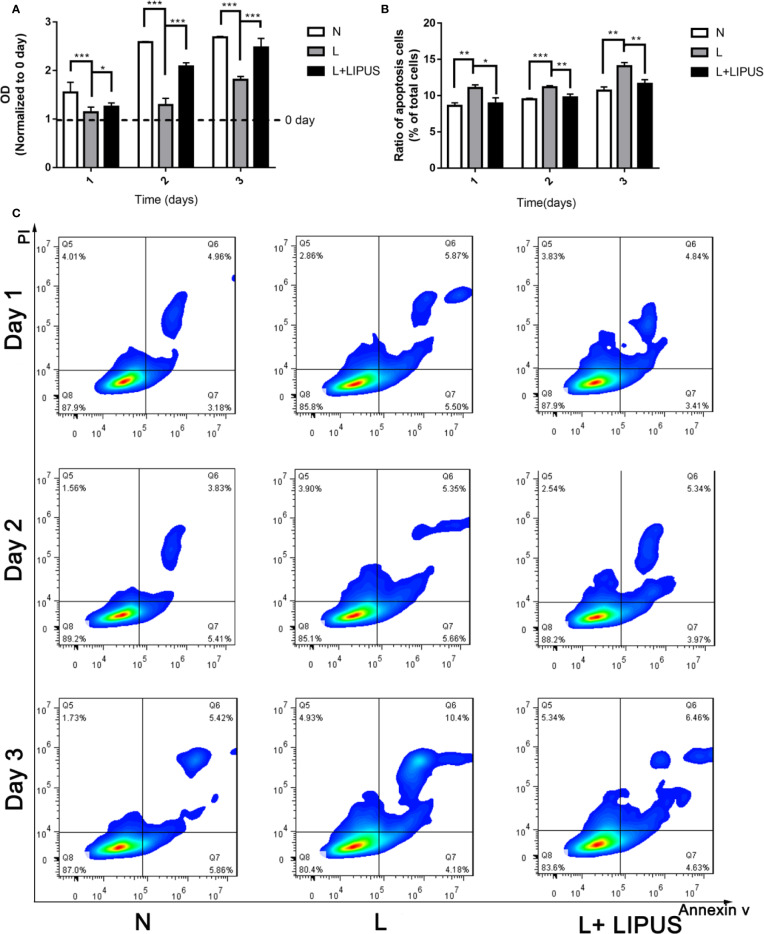
Low-intensity pulsed ultrasound (LIPUS) promotes the proliferative capacity of chondrocytes under hypoxia and inhibits apoptosis. **(A)** Proliferative capacity of chondrocytes under normal (N) conditions, hypoxic conditions (L), and following LIPUS stimulation (L + LIPUS). **(B, C)** Ratio of apoptotic cells in chondrocytes treated under normal (N) conditions, hypoxic conditions (L), and following LIPUS stimulation (L + LIPUS). Data are shown as the mean ± SD; *p < 0.05, **p < 0.01, ***p < 0.001. Experiments were performed at least three times.

First, we verified that LIPUS has a beneficial effect on chondrocytes grown under hypoxic conditions. We showed that the proliferative capacity of mandibular chondrocytes was significantly suppressed under hypoxic conditions and that LIPUS application partially restored cell growth (**Figure 1A**). The apoptotic rate of mandibular chondrocytes also significantly increased under hypoxic conditions compared to that in controls; however, hypoxia-induced apoptosis was ameliorated upon treatment with LIPUS (14.58% of cells were apoptotic in the hypoxia group at day 3 *vs.* 11.09% in the LIPUS-treated group and 11.28% in the control group; **Figures 1B, C**).

### LIPUS Treatment Increases Type II Collagen Expression and Promotes Chondrogenic Capacity

**Figure 2:**
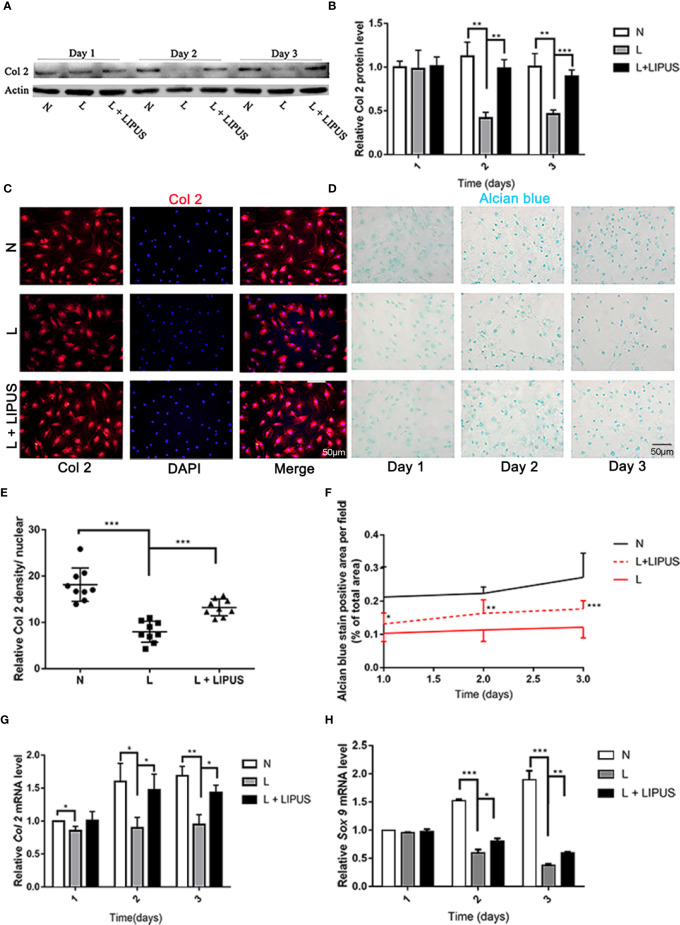
Chondrogenic capacity is upregulated by low-intensity pulsed ultrasound (LIPUS). **(A, B)** Western blot analysis of type II collagen (Col2) protein levels in chondrocytes under normal (N) conditions, hypoxic conditions (L), and following LIPUS stimulation (L + LIPUS). **(C, E)** Immunofluorescence staining of Col2 in chondrocytes from the three groups. **(D, F)** Alcian blue staining of mucin sulfate in chondrocytes from the three groups. **(G, H)** mRNA expression of *Col2* and *Sox9* in chondrocytes from the three groups. Data are shown as the mean ± SD; *p < 0.05, **p < 0.01, ***p < 0.001. Experiments were performed at least three times.

Type II collagen expression is positively related with chondrogenic capacity (Gale et al., 2019) and was previously shown to significantly decrease under hypoxic conditions (Anderson et al., 2016). Similarly, we found that hypoxia significantly decreased the expression of type II collagen (Col2) protein in mandibular chondrocytes compared to that in controls and cells treated with LIPUS (**Figures 2A, B**). Immunofluorescence experiments confirmed that type II collagen expression was significantly reduced in mandibular chondrocytes grown under hypoxic conditions and that treatment with LIPUS restored its expression by day 3 (**Figures 2C, E**).

Mucin sulfate, a major component of cartilage tissue, can be detected by Alcian blue staining (Kiani et al., 2002). We found that the area stained with Alcian blue decreased under hypoxic conditions compared to that in controls (**Figures 2D, F**), confirming that hypoxia suppresses the chondrogenic capacity of mandibular chondrocytes. LIPUS application partially rescued this impaired chondrogenic capacity (**Figures 2D, F**). We also examined the mRNA expression of *Col2* and *SOX9*, and the results were consistent with those for the protein expression level (**Figures 2G, H**).

### LIPUS Treatment Decreases IL-6/MMP-3 Levels and Increases TIMP-1 Levels

**Figure 3:**
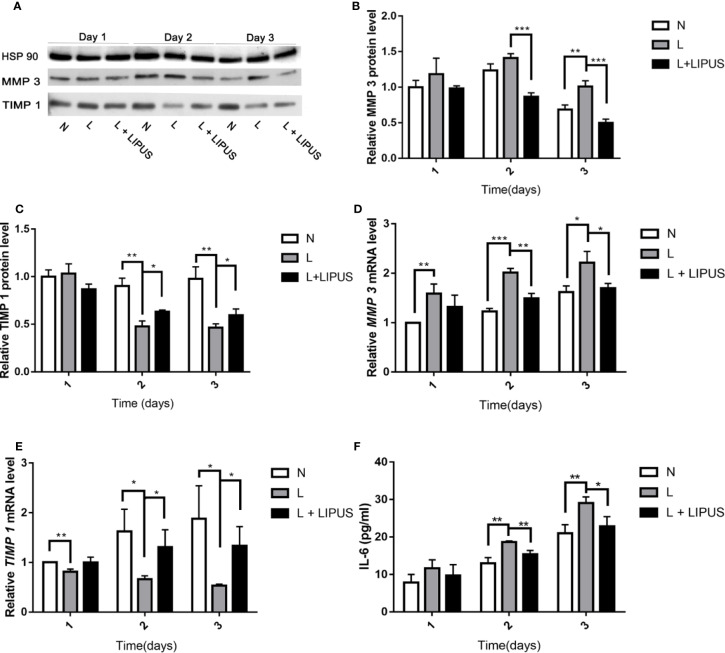
Low-intensity pulsed ultrasound (LIPUS) decreases MMP-3 levels and increases TIMP-1 and IL-6 levels. **(A–C)** Western blot analysis of HSP90, MMP-3, and TIMP-1 protein levels in chondrocytes under normal (N) conditions, hypoxic conditions (L), and following LIPUS stimulation (L + LIPUS). **(D, E)** Expression of *MMP-3* and *TIMP-1* mRNA in chondrocytes from the three groups. **(F)** IL-6 levels in chondrocytes from the three groups. Data are shown as the mean ± SD; *p < 0.05, **p < 0.01, ***p < 0.001. Experiments were performed at least three times.

Next, we evaluated the severity of chondrocyte injury by determining the expression of MMP-3 and TIMP-1. MMP-3 and TIMP-1 protein levels were slightly increased at day 1 under hypoxic conditions. Although LIPUS application slightly increased TIMP-1 expression and decreased MMP-3 expression in mandibular chondrocytes, the levels were not significantly different from those found in hypoxia-treated cells (**Figures 3A–C**). However, by days 2 and 3, MMP-3 protein expression was significantly upregulated under hypoxic conditions. LIPUS application reduced MMP-3 expression in mandibular chondrocytes cultured under hypoxic conditions. In addition, the expression of *MMP-3* mRNA was significantly increased and *TIMP-1* mRNA expression was significantly decreased in mandibular chondrocytes grown under hypoxic conditions compared to those in controls (N group; **Figures 3D, E**). Meanwhile, *MMP-3* mRNA levels decreased, and *TIMP-1* mRNA levels increased, following LIPUS application (**Figures 3D, E**). We also investigated IL-6 protein levels using ELISA. Hypoxia increased IL-6 levels in mandibular chondrocytes compared to those in controls, whereas LIPUS application significantly decreased levels of this marker (**Figure 3F**).

### LIPUS Treatment Upregulates HIF-1α Levels and Downregulates HIF-2α Expression Under Hypoxic Conditions

**Figure 4:**
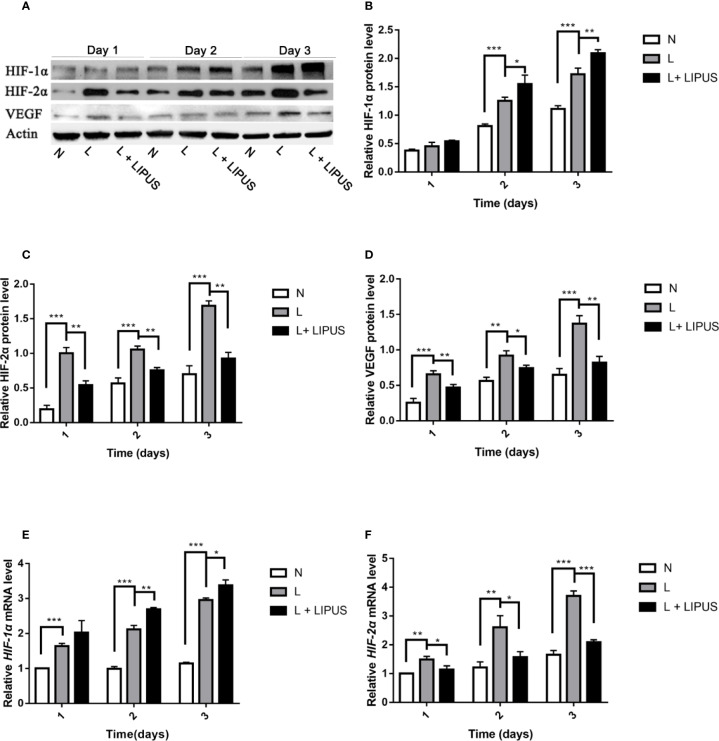
Low-intensity pulsed ultrasound (LIPUS) increases HIF-1α expression and decreases HIF-2α expression. **(A–D)** Western blot analysis of HIF-1α, HIF-2α, and VEGF protein levels in chondrocytes under normal (N) conditions, hypoxic conditions (L), and following LIPUS stimulation (L + LIPUS). **(E, F)** Expression of *HIF-1α* and *HIF-2α* mRNA in chondrocytes from the three groups. Data are shown as the mean ± SD; *p < 0.05, **p < 0.01, ***p < 0.001. Experiments were performed at least three times.

The HIF pathway involves two major factors, namely HIF-1α and HIF-1β. This pathway is associated with oxygen tension and becomes activated under hypoxic conditions (Bouaziz et al., 2016). HIF-1α in particular is an oxygen-regulated subunit that regulates the expression of related hypoxia-induced genes (Ke and Costa, 2006). We found that the expression of both HIF-1α and HIF-2α proteins was upregulated in mandibular chondrocytes cultured under hypoxic conditions (**Figure 4A**), in line with previous studies (Zhang et al., 2015). The expression of HIF-1α was further enhanced after LIPUS application at days 2 and 3 (**Figure 4B**). HIF-2α expression was also increased under hypoxic conditions but was decreased following LIPUS application (**Figure 4C**). Consistently, the expression of VEGF, a downstream target of the HIF pathway, increased in mandibular chondrocytes grown under hypoxic conditions and decreased following LIPUS application (**Figure 4D**). The expression of *HIF-1α* and *HIF-2α* was also upregulated at the mRNA level in response to hypoxic conditions, whereas LIPUS application increased *HIF-1α* and decreased *HIF-2α* mRNA expression (**Figures 4E, F**). Together, our results indicate that LIPUS exerts its beneficial effects on chondrocytes cultured under hypoxia by up-regulating HIF-1α and down-regulating HIF-2α.

### LIPUS Protects Against Chronic Sleep Deprivation-Induced Chondrocyte Damage in a Rat Model

**Figure 5:**
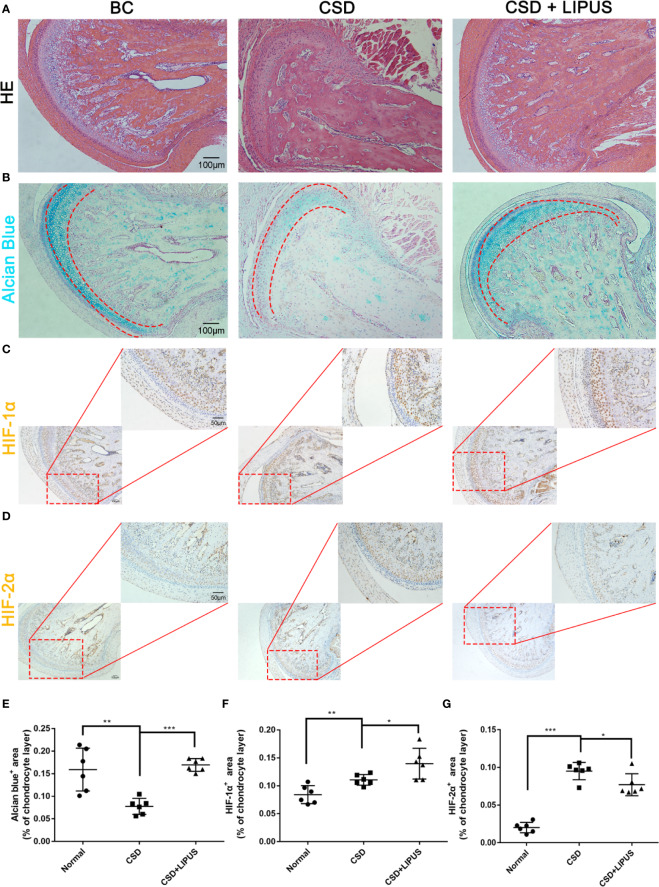
Low-intensity pulsed ultrasound (LIPUS) partially repairs condylar processes *via* the HIF pathway in a rat model of chronic sleep deprivation. **(A)** Histological structures of condylar processes in blank control rats (BC) compared to those in a rat model of chronic sleep deprivation (CSD) and rats treated with LIPUS (CSD + LIPUS). **(B, E)** Alcian blue staining of sections obtained from BC, CSD, and CSD + LIPUS rats. **(C, F)** Both 10× and 20× images are presented to show the expression of HIF-1α in the three groups. **(D, G)** Both 10× and 20× images are presented to show the expression of HIF-2α in the three groups. *N* = 6 per group. Data are shown as the mean ± SD; *p < 0.05, **p < 0.01, ***p < 0.001. Experiments were performed at least three times.

**Figure 6:**
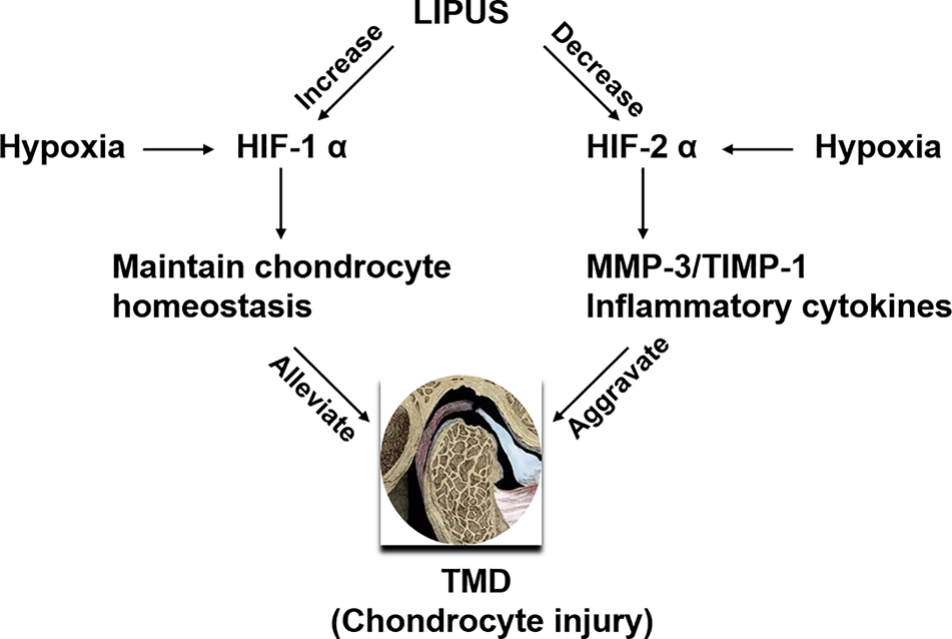
Schematic of the proposed benefits of low-intensity pulsed ultrasound (LIPUS) treatment for temporomandibular joint disorders (TMDs). LIPUS protects chondrocytes from hypoxia-induced injury by enhancing HIF-1α expression and downregulating HIF-2α expression.

We previously reported that LIPUS protects chondrocytes from damage in a rat model of CSD by reducing MMP-3 levels and increasing osteoprotegerin levels (Liang et al., 2019); however, we did not assess changes in the HIF pathway. Here, our histological observations indicated that the chondrocyte layer was partially impaired in the TMJ of CSD rats compared to that in control rats and that LIPUS treatment could maintain the normal physiological structure of the condylar process (**Figure 5A**). In agreement with our *in vitro* findings, LIPUS treatment restored the chondrogenic ability of the TMJ in these rats (p < 0.001; **Figures 5B, E**). Moreover, both HIF-1α and HIF-2α were significantly upregulated in the chondrocyte layer of CSD rats (**Figures 5C, D, F, G**), indicating that the HIF pathway was activated. The original images of HIF-1α and HIF-2α staining were showed in **Supplementary Figure 3**. After LIPUS treatment, HIF-1α levels were further increased (p < 0.05), whereas HIF-2α levels were decreased (p < 0.05), compared to those in untreated CSD rats. Based on our results, we speculated that LIPUS could efficiently alleviate hypoxia induced chondrocytes injury through reducing inflammation and maintaining chondrogenic capacity. The beneficial effect of LIPUS was possibly associated with HIF pathway (**Figure 6**).

## Discussion

We examined whether LIPUS application could alleviate hypoxia-induced chondrocyte injury in TMDs. Results showed that LIPUS could restore the proliferative capacity of rat mandibular chondrocytes cultured under hypoxic conditions and reduce their apoptotic rate. LIPUS application also rescued the chondrogenic capacity of rat mandibular chondrocytes and reduced levels of the inflammatory cytokine IL-6 and MMPs (MMP-3). Moreover, it enhanced the expression of HIF-1α, which is typically induced by hypoxia, and reduced the expression of HIF-2α. Our *in vitro* results were confirmed *in vivo* using a rat model of CSD. Together, our results indicate that the beneficial effect of LIPUS might partly be associated with the HIF pathway.

Previous studies indicate that local hypoxia in articular cartilage can induce chondrocyte apoptosis (Huang et al., 2017), and this was confirmed in our *in vitro* experiments. We further found that LIPUS treatment could not only decrease the apoptotic rate of mandibular chondrocytes but also increase their proliferative capacity, indicating its beneficial effect on repairing chondrocyte injury caused by hypoxia.

Hypoxia can also affect the levels of major components of the extracellular matrix (e.g. aggrecan and type II collagen) that are required to maintain the normal physiological functions of cartilage (Zhu et al., 2019). Indeed, it has been shown that decreased expression of aggrecan and type II collagen can damage the normal biological structure of joints (Liu et al., 2019). We found that type II collagen expression was decreased and that the mucin-positive area was reduced upon exposure to hypoxic conditions both *in vivo* and *in vitro*, confirming that chondrocyte matrix production capability was impaired by hypoxia. We also showed that LIPUS application could efficiently restore the chondrocyte matrix production capability, as indicated by the increase in type II collagen expression and the enlargement of the mucin-positive area.

Inflammatory cytokines and MMP-3 have been shown to accumulate under hypoxic conditions (O'rourke et al., 2011; Kwon et al., 2017). IL-6 is a pro-inflammatory cytokine that plays a vital role in the destruction of articular cartilage (Haseeb et al., 2017), and MMP-3 can accelerate degradation of the cartilage matrix by cleaving multiple extracellular matrices (Dai et al., 2019). We found that MMP-3 and IL-6 levels were increased under hypoxic conditions, while TIMP-1 levels decreased, highlighting the impaired chondrocyte function. In addition, LIPUS treatment partially restored MMP-3 and TIMP-1 expression, and reduced IL-6 levels.

Previous studies indicated that hypoxia could upregulate the HIF pathway and might subsequently initiate a pathological process or protective biofunction in cartilage (Bouaziz et al., 2016a; Laddha and Kulkarni, 2019). Indeed, the HIF-1 pathway was previously shown to play a vital role in the maintenance of chondrocyte homeostasis and chondrogenic capacity (Dunwoodie, 2009). In particular, HIF-1α can regulate chondrogenesis by upregulating the expression of SOX9, Col2, and aggrecan (Zhang et al., 2015). Moreover, activated HIF-1α signaling can block the TCF4–β-catenin axis, thus inhibiting cartilage damage (Bouaziz et al., 2016a). Meanwhile, HIF-2α is typically regarded as a central catabolic transcription factor in osteoarthritis that aggravates osteoarthritic cartilage destruction by increasing matrix-degrading enzyme levels (Patel and Simon, 2008; Ryu et al., 2011). Activated HIF‐2α has also been shown to induce chondrocyte apoptosis *via* the Fas pathway, thus aggravating osteoarthritis-associated cartilage destruction (Ryu et al., 2012). HIF-2α might also directly activate its target gene *IL-6* and upregulate MMP-3 levels to promote cartilage destruction (Ryu et al., 2014). Therefore, HIF-1α and HIF-2α play almost contradictory roles in maintaining chondrocyte homeostasis.

The pathological factors (such as psychological factors and occlusal factors) of TMD are complex, and a CSD-induced cartilage injury has been reported as a TMD animal experimental model (Ma et al., 2014; Ding et al., 2017). We found that both HIF-1α and HIF-2α levels were significantly increased under hypoxic conditions. LIPUS treatment increased HIF-1α levels but decreased HIF-2α levels *in vitro*, indicating that it regulates the HIF pathway. These findings were also confirmed in a rat model of CSD; that is, HIF-1α expression was increased and HIF-2α expression was decreased following LIPUS treatment. In addition, the disordered joint structure induced by hypoxia was repaired after LIPUS treatment. Overall, our results show that the HIF pathway is important for the pathological process that underlies hypoxia-induced TMJ injury and that LIPUS treatment can affect both HIF-1α and HIF-2α levels in mandibular chondrocytes.

LIPUS is a form of mechanical microwave stimulation, with a wide range of clinical applications including healing bone fractures, regulating the differentiation of mesenchymal stem cells (Romano et al., 2009; Ling et al., 2017), and recovering the osteogenic capacity of osteoblastic cells (Kusuyama et al., 2019). Indeed, the beneficial effects of LIPUS in alleviating chronic pain (including osteoarthritis) and promoting bone fracture healing are well known (Wang et al., 2019). Despite this, its influence on chondrocytes cultured under hypoxia has seldom been reported. Hypoxia activates the HIF pathway, leading to the accumulation of multiple inflammatory cytokines and MMPs (Gao et al., 2015), aggravating the associated pathological process. We found that LIPUS can efficiently mitigate hypoxia-induced chondrocyte injury in the TMJ and that this bio-function is partly associated with the HIF pathway.

This study showed that LIPUS treatment can rescue impaired mandibular chondrocytes both *in vitro* and *in vivo*. The biological function of mandibular chondrocytes, as well as their proliferation capacity, was recovered following LIPUS stimulation. This treatment also suppressed the hypoxia-induced accumulation of inflammatory cytokines and MMPs. Based on these results, we suggest that the beneficial effect of LIPUS is partly associated with the HIF pathway. It has been reported that LIPUS targets miR-31-5p to regulate HIF-1α. However, the mechanism underlying LIPUS regulation of HIF-2α has rarely been reported (Costa et al., 2019). Thus, the mechanism by which LIPUS influences HIF pathways is still unclear and requires further study. Overall, our results indicate that LIPUS could be an effective strategy to treat hypoxia-induced chondrocyte damage, with useful clinical applications for TMDs.

## Data Availability Statement

The datasets generated for this study are available on request to the corresponding author.

## Ethics Statement

The animal study was reviewed and approved by Ethics Committee of Beijing Stomatological Hospital (Approval code: KQYY-201610-001).

## Author Contributions

This study was supported and designed by WG and JL. Most experiments were performed by TY and CL. The manuscript was written by TY. LC contributed to the analysis of the results.

## Funding

This study was supported by the National Natural Science Foundation of China (Grant No. 61571311) (WG) and the High-Level Health Technical Personnel in Beijing Preferred Foundation (2015-3-091) (WG).

## Conflict of Interest

The authors declare that the research was conducted in the absence of any commercial or financial relationships that could be construed as a potential conflict of interest.
