# Supplementary material for: Low-Intensity Pulsed Ultrasound Alleviates Hypoxia-Induced Chondrocyte Damage in Temporomandibular Disorders by Modulating the Hypoxia-Inducible Factor Pathway
Source: Front Pharmacol. 2020 May 14;11:689. doi: 10.3389/fphar.2020.00689 (PMC7240017; doi:10.3389/fphar.2020.00689)
Supplement: Supplementary file 1 [file DataSheet_1.doc]

Supplementary figure 1: The identification of chondrocyte


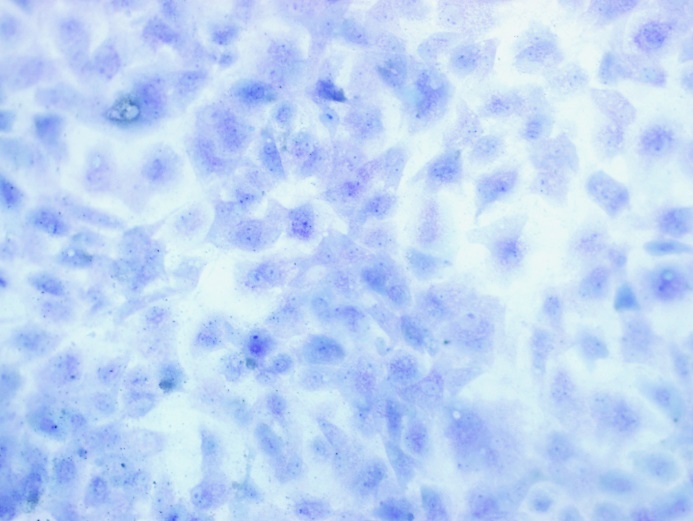


Toluidine blue staining which could specifically recognize glycosaminoglycan, was used to identify the chondrocyte. As shown, cell cytoplasm was stained as bluish violet and cell nuclei was dyed mazarine blue (x 400).

Supplementary figure 2: LIPUS intervention method schematic diagram


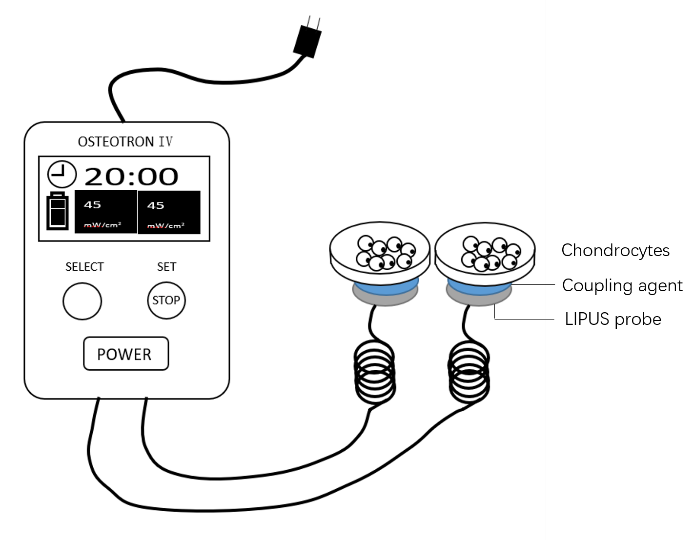


The LIPUS therapy generated with OSTEOTRON IV ultrasonic therapy device (ITO Ultrashort Wave Co. Ltd., Tokyo, Japan) was described as follow: 45 mW/cm2 ultrasonic intensity, 1.0 MHz ultrasonic frequency and 200 μs pulse width. When presenting the LIPUS treatment, coupling agent was used to connect the LIPUS probe and the bottom of the culture dish. LIPUS stimulation was lasted for 20min per day.

Supplementary figure 3: Original images of HIF-1α and HIF-2α IHC staining in rats


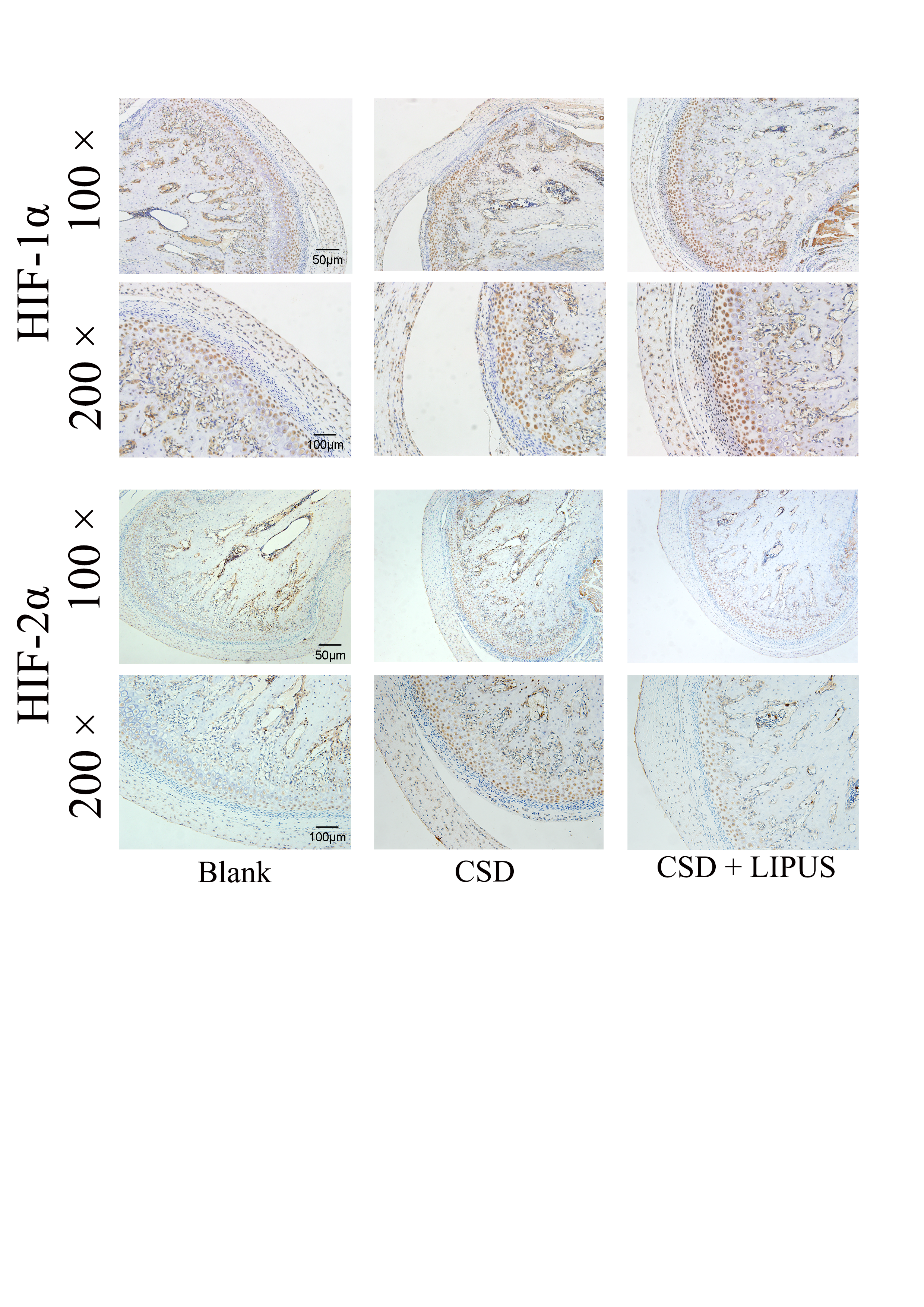


Both 100 × and 200 × magnification images of HIF-1α and HIF-2α IHC staining in experimental animals were showed. Six animals were randomly separated into three groups. The condylar was harvested, made to slices and observed using microscope.
